# Strategies to improve deep learning-based salivary gland segmentation

**DOI:** 10.1186/s13014-020-01721-1

**Published:** 2020-12-01

**Authors:** Ward van Rooij, Max Dahele, Hanne Nijhuis, Berend J. Slotman, Wilko F. Verbakel

**Affiliations:** Department of Radiation Oncology, Cancer Center Amsterdam, Vrije Universiteit Amsterdam, Amsterdam UMC, de Boelelaan 1117, Amsterdam, The Netherlands

**Keywords:** Artificial intelligence, Deep learning, Salivary glands, Segmentation

## Abstract

**Background:**

Deep learning-based delineation of organs-at-risk for radiotherapy purposes has been investigated to reduce the time-intensiveness and inter-/intra-observer variability associated with manual delineation. We systematically evaluated ways to improve the performance and reliability of deep learning for organ-at-risk segmentation, with the salivary glands as the paradigm. Improving deep learning performance is clinically relevant with applications ranging from the initial contouring process, to on-line adaptive radiotherapy.

**Methods:**

Various experiments were designed: increasing the amount of training data (1) with original images, (2) with traditional data augmentation and (3) with domain-specific data augmentation; (4) the influence of data quality was tested by comparing training/testing on clinical versus curated contours, (5) the effect of using several custom cost functions was explored, and (6) patient-specific Hounsfield unit windowing was applied during inference; lastly, (7) the effect of model ensembles was analyzed. Model performance was measured with geometric parameters and model reliability with those parameters’ variance.

**Results:**

A positive effect was observed from increasing the (1) training set size, (2/3) data augmentation, (6) patient-specific Hounsfield unit windowing and (7) model ensembles. The effects of the strategies on performance diminished when the base model performance was already ‘high’. The effect of combining all beneficial strategies was an increase in average Sørensen–Dice coefficient of about 4% and 3% and a decrease in standard deviation of about 1% and 1% for the submandibular and parotid gland, respectively.

**Conclusions:**

A subset of the strategies that were investigated provided a positive effect on model performance and reliability. The clinical impact of such strategies would be an expected reduction in post-segmentation editing, which facilitates the adoption of deep learning for autonomous automated salivary gland segmentation.

## Background

Target volume and organ-at-risk (OAR) delineation are fundamental steps in the radiotherapy treatment planning process. However, they are time and labor intensive and prone to inter and intra-observer variation. Automated deep-learning (DL) based delineation, in particular of OARs, has been investigated to address the challenges associated with manual delineation [1, 2]. Although results have generally been promising, even for anatomically complex regions like the head-and-neck [3], further improvements are desirable for various reasons, including enabling more reliable OAR dose-volume and toxicity data, and reducing manual checking and editing during on-line adaptive radiotherapy. We investigate the potential for improvement in DL segmentation, taking the salivary glands as a paradigm. These OARs have long been recognized as important in head-and-neck radiotherapy and insufficient sparing can result in toxicity and reduced quality of life [4]. Contouring them can be challenging for technical reasons like low grey-scale contrast with surrounding tissue and variation in shape, and clinical reasons like inadequate anatomical knowledge. Although performance of DL-based delineation (DLD) for salivary glands is such that it can be used to create clinically acceptable treatment plans [5], it is by no means perfect—there have been no reports of salivary gland DLD models reaching performances above a Sørensen–Dice coefficient of ~ 90% [3]. Also, most studies looking at improvements to DLD usually only investigate a single strategy. In contrast, we wanted to compare the effect of multiple individual strategies, and then combine the strategies that proved useful in order to see if there was any obvious synergism. All strategies that were investigated can be applied to any given DLD model.

## Methods

DL model improvement was investigated for left parotid and submandibular glands (PG/SMG) using 3D CT data (acquired on GE discovery 590RT, helically scanned, 512 × 512 pixels per slice) from head-and-neck cancer (HNC) treatments. There were 683/564 clinically contoured left PGs/SMGs. Whenever the right PG/SMG was available, it was flipped and added to the dataset (based on the assumption of symmetry [6]), resulting in a total of 1365/1128 PGs/SMGs. The PG/SMG voxel spacing was 1.00 ± 0.08 mm by 1.00 ± 0.08 mm by 2.49/2.48 ± 0.14/0.16 mm (± indicating standard deviation). Clinical delineation was done by > 10 HNC radiation oncologists and supervised residents. No additional curation was performed.

**Fig. 1:**
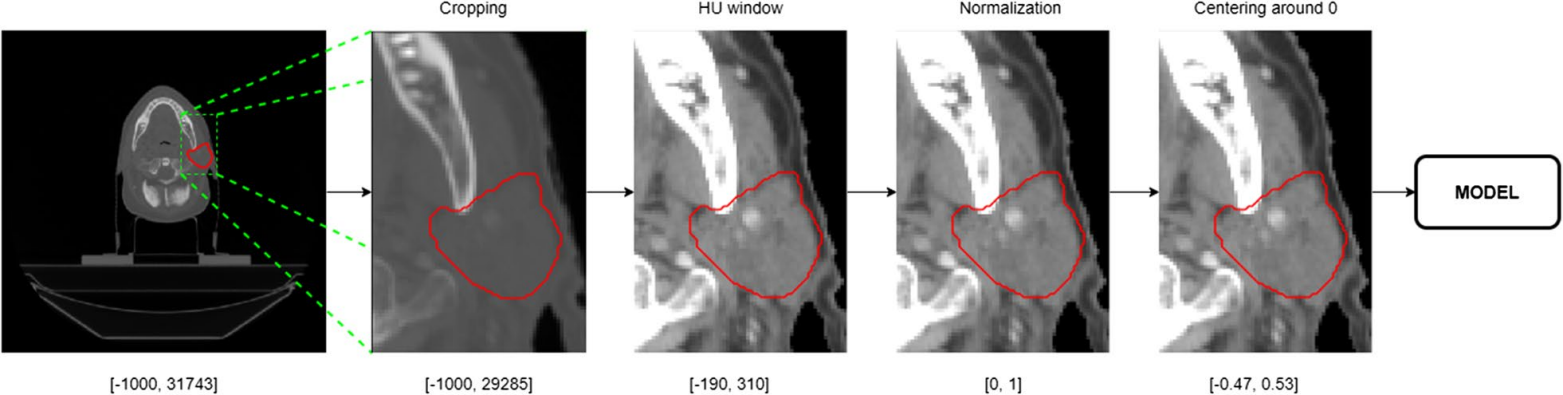
Overview of the preprocessing steps for the base set-up for a random PG. The clinical contour is depicted in red. The corresponding value ranges are given below the images. The steps are identical for the SMG

A base DL set-up was used, to which specific strategies were applied. For example, the base dataset was used every time when the applied strategy had no influence on the dataset and the base cost function was used every time the applied strategy had no influence on the cost function. This was done to keep the effects from different experiments as comparable as possible. The base dataset comprised 120 cases. Even with this size, the total training time for all the models in this study was ~ 30 days, which is why using the full data set of 1365/1128 PGs/SMGs in all experiments would not have been feasible. For each experiment, 5/6 of the dataset was randomly selected to be the train set and the remaining 1/6 of the dataset comprised the test set. The specific sizes of the sets varied, because for some experiments the base dataset was supplemented with additional cases (e.g. when testing the effect of the amount of training data). Six-fold cross-validation was applied so that every fold contained samples not appearing in the test set in other folds; thus, every case was in the test set at some point during an experiment. The base-model was a fully convolutional network [7], based on the 3D U-net [5, 8], with dropout [9] applied to all convolutional layers, the Sørensen-Dice coefficient (SDC) as cost function, Adam [10] as the optimizer and early stopping was applied to prevent overfitting; training was stopped when improvement for a separate validation set was < 0.001 for at least 4 epochs. In all experiments, the validation set comprised a random 10% of the train set, which was not used for training the model. Even though this might have caused some models to stop training prematurely, it was assumed that the main experimental effect would be observed. SDC is defined as:

$$SDC = \frac{2tp}{{2tp + fp + fn}} ,$$

where *tp*, *fp* and *fn* are the number of true positive, false positive and false negative voxels respectively. The models were built with Keras (https://keras.io/) on top of TensorFlow (https://www.tensorflow.org/). The base preprocessing steps consisted of cropping a region-of-interest (ROI; 64 × 64 × 32/96 × 64 × 64 voxels for SMG/PG) centered on the OAR to limit memory usage, applying a Hounsfield Unit (HU) window (− 75 to 175 and − 190 to 310 for SMG and PG respectively) to remove extreme values and increase contrast and finally normalizing the data, because convergence is reached more easily when all input variables have the same range. All values were normalized to a range of [0,1] after which the mean was subtracted, effectively centering the data around 0 (Fig. 1). The evaluation of each experiment was done using SDC and 3D Hausdorff distance (HD; largest minimal distance from any point from set *a* to any point from set *b*; units of voxel space). Calculations were done on a single GeForce GTX 1080ti GPU. Hyperparameter values were chosen based on prior non-exhaustive hyperparameter tuning. The various experiments change different elements of the base-model: data quantity, data quality, model training and how to use the model for inference.

### Data quantity

#### Effect of set size

More data usually means better model performance, but linear increases in amounts of data often result in exponentially decreasing increases in model performance [11]. Since data of (high-quality) clinical delineations is often hard to come by, it is relevant to investigate whether the effort to collect it is worthwhile. Therefore, we trained the base-model multiple times with different set sizes: PG = 60–1320 and SMG = 60–1080 in increments of 60. The small set size of 60 was introduced to allow for a more complete examination of the relationship between set-size and model performance.

#### Effect of traditional data augmentation

Data augmentation can be used to substitute for more original data [12]. This consists of applying operations to original images in order to create new training images. We randomly applied flipping, rotation in all angles and adding Gaussian noise (µ = 0, σ = 15) to 60–600 random cases from the base train set (one case can be augmented multiple times) in increments of 60. These cases were then added to the training data.

#### Effect of domain-specific data augmentation

**Fig. 2:**
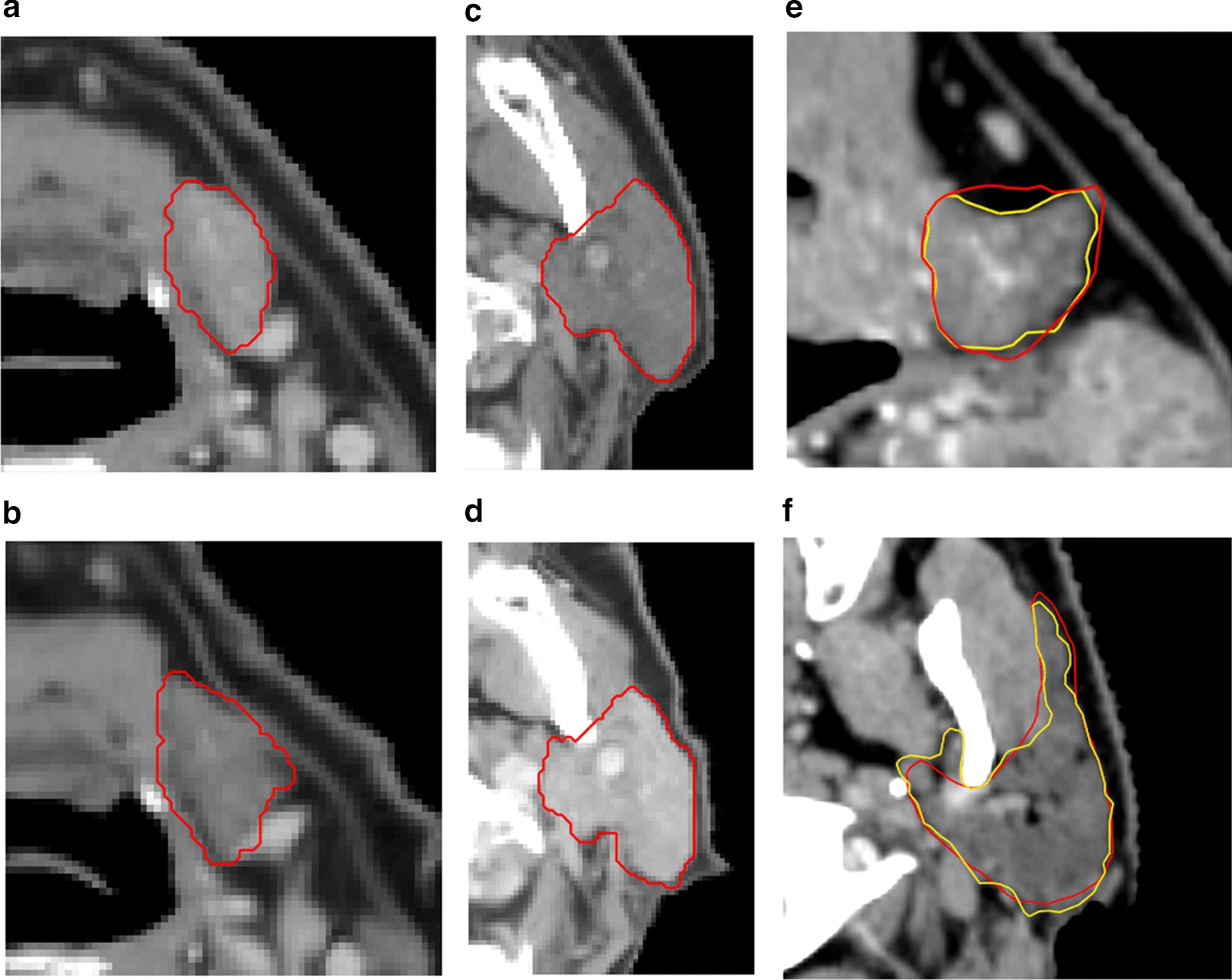
Example of an original image (**a**/**c**) and its domain-specific augmented version (**b**/**d**) for a SMG (**a**/**b**) and a PG (**c**/**d**) and examples of clinical (red) vs. curated (yellow) contours for SMG/PG (**e**/**f**)

In the case of CT data, traditional data augmentation methods generate images which can be very easily distinguished from the original dataset (e.g., an original CT scan is never flipped without reason). Therefore, other data augmentation techniques may be more useful to simulate cases that might be expected to be found in the original dataset. We call this approach domain-specific data augmentation. More technically, the aim is to increase the inherent data variance rather than introduce new forms of variance. In order to do this, three operations were applied for each augmented case: elastic deformation simulating different anatomies ([13]; α = 38/58, σ = 3.8/5.8, affine α = 3.8/5.8 for SMG/PG), shifting patient densities by adding a value to all voxels in the body contour and shifting OAR densities by adding a value to all voxels in the OAR contour. Shifting was done by drawing a random value from a Gaussian distribution (µ = 0/0, σ = 100/30 respectively). Examples are shown in Fig. 2a–d.

### Data quality

#### Effect of data curation

Clinical OAR delineations often contain inaccuracies (e.g. PG delineation containing part of the mandible, extending into skin, containing muscle tissue or missing part of the organ). This may be due to insufficient attention to detail, low contrast with surrounding tissue, (metal) CT-scan artifacts or patient-specific organ deformation. Usually, such inaccuracies are relatively minor and may not influence treatment plan quality [14] (clinicians being aware of the small effect on dose distributions may itself be a cause of the inaccuracies), but they can hinder the DL model from learning to properly recognize the structure. Therefore, we investigated the effect of meticulous (voxel-level) curation of the clinical contour. A single observer performed this task in order to reduce variability. Curation took ~ 1 h per gland, whereas clinical delineation usually takes a few minutes for the PG/SMGs. Examples of the initial clinical contour and its curated version can be seen in Fig. 2e/f. For this preliminary analysis, 24 SMGs/PGs were curated. Training and testing on clinical/curated cases was compared. Because of the small sample size, we repeated the tests after domain-specific augmentation of the training data to the size of the base train set (n = 90). Each cross-validation fold was run 6 times, to account for random weight initialization and make the results more reliable. This was facilitated by the small set size.

### Model training

#### Effect of cost function

Volumetric SDC can be relatively insensitive to substantial surface-level errors, because their volume is often small compared to the entire structure. Because of this, it may be that model training stops earlier than desirable when the volumetric similarity between the clinical and DL delineation overpowers the influence of surface distance errors. In order to increase the impact of surface errors during training, we ran the base experiment using a volumetric SDC cost function, while decreasing the influence of true positive voxels by factor 2 (SDC(0.5)) and 20 (SDC(0.05)). We also trained using a combined SDC + HD cost function:

$$Cost = SDC + \frac{HD}{{0.33x}} ,$$

where *x* is the ROI diagonal (maximum possible HD), which forces the HD to a range of 0–1. The value of 0.33 limiting *x* was chosen empirically to make sure *x* did not nullify the influence of HD. SDC + HD training was only done for the SMG, because prior tests showed that calculating HD for the PG during training exceeded the GPU memory available to us (PG ROI was larger than the SMG ROI).

### Inference

#### Effect of patient-specific Hounsfield unit windowing

In prior tests we found that using a patient-specific HU window for inference, instead of a set window across the entire patient dataset, might increase model performance and reliability. To properly validate if this effect holds for different models, we searched for the ‘best’ patient-specific window (center: − 100–400 in increments of 10, width: 100–1000 in increments of 50) used to preprocess the data, and picked the window that provided the highest SDC. We use the term ‘best window’ loosely, because an exhaustive search of all possible windows was not performed. This was repeated for 6 models with different test sets, in keeping with the sixfold cross-validation in other experiments. Since only 12 models had to be trained for this experiment (6 for each gland), we could use the entire dataset, increasing the outcome’s validity.

#### Effect of ensembles

Ensemble methods consist of averaging multiple models’ predictions through some voting scheme (i.e. by defining a voxel as positive only when a minimum number of models, the cut-off, predict it to be positive), to provide more accurate predictions than that of a single model [15]. We tried an ensemble of 11 models and utilized the random initialization of model parameters to generate them. Different parameter initializations can converge at different local optima. Therefore, different models can complement each other. The ensembles’ performance was compared to the average performance of all stand-alone models. This process was done 6 times in keeping with the sixfold cross-validation in other experiments.

#### Effect of all positive interventions on the entire dataset

Finally, the strategies that had a positive effect on model performance/reliability were combined in a final model. This model was compared to a model trained with only the maximum set size as the intervention.

## Results

**Fig. 3:**
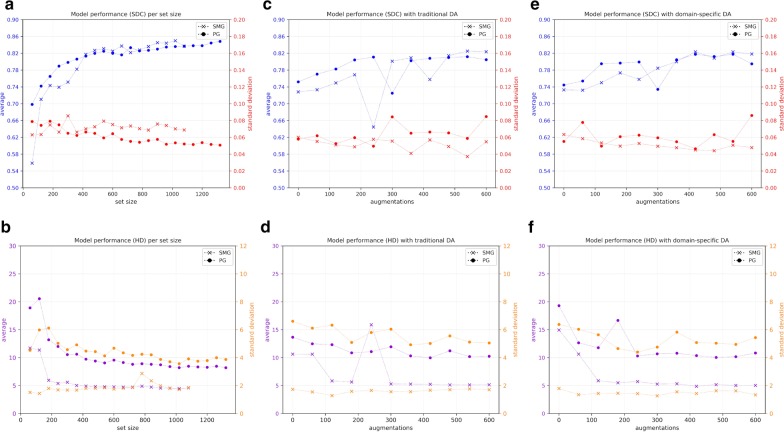
Model performance/reliability measured by SDC/HD per set size (**a**/**b**), per number of augmented images with traditional data augmentation (**c**/**d**) and per number of augmented images with domain-specific augmentation (**e**/**f**)

**Fig. 4:**
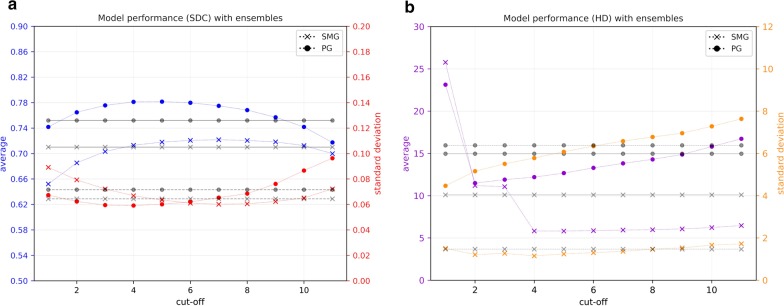
Model performance/reliability measured by SDC/HD (**a**/**b**) with ensemble methods, including the effect of different cut-offs. Grey lines show average (-)/standard deviation(–) of SDC/HD for all stand-alone models in this experiment

**Fig. 5:**
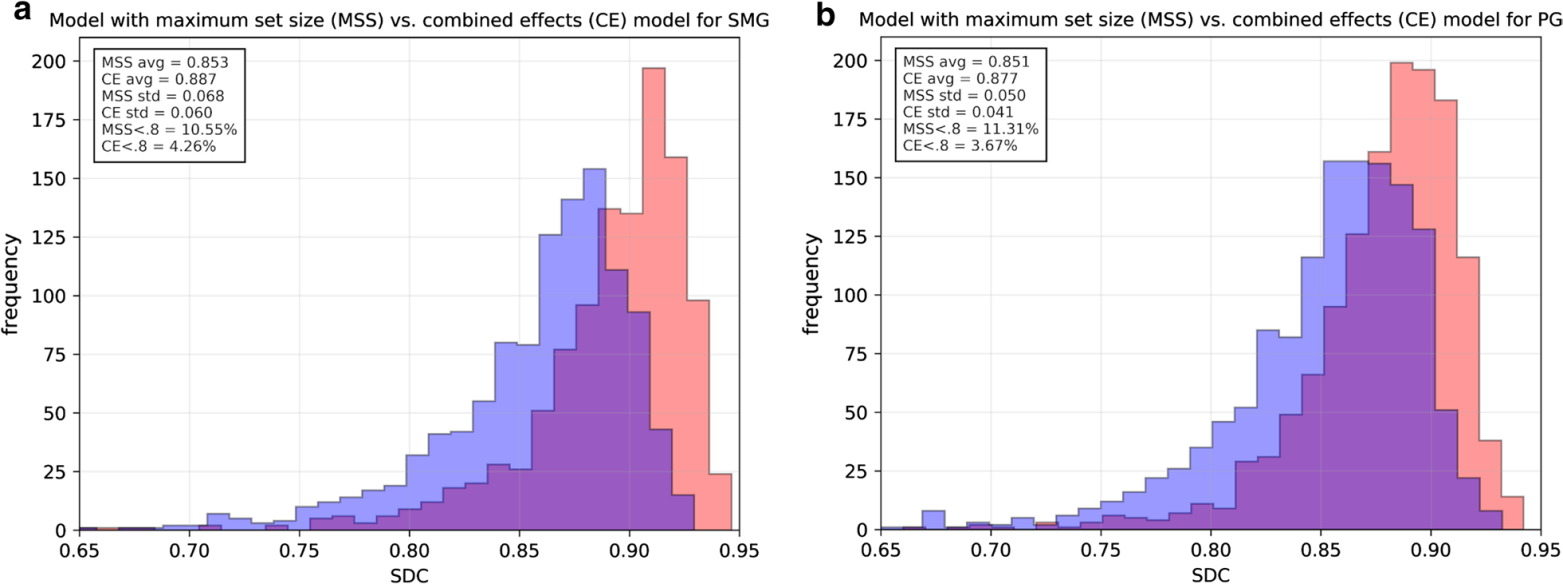
Model trained with the maximum set size (MSS; blue) versus an ensemble of models trained with the maximum set size, doubled in size by domain-specific data augmentation and with patient-specific windowing applied (CE; red) for SMG/PG

The results can be seen in Fig. 3, Table 1 and Fig. 4. The presented results are the combined results of all cross-validation folds. They demonstrate the following. Increasing set size provides an exponentially decreasing increase in model performance for PG/SMG and an increase in model reliability for the PG, but not SMG (Fig. 3a/b). Overall, both types of data augmentation appear to have a similar positive effect on model performance but not reliability for both OARs (Fig. 3c–f). However, the trend is not completely stable, especially not for the traditional data augmentation. Training on curated rather than clinical contours, with or without augmentation, did not yield better results for either OAR (Table 1). None of the cost functions gave better results than the SDC for both OARs (Table 1). Using a patient-specific HU window had a positive effect on model performance/reliability for both OARs (Table 1). Model ensembles appear to provide better and more reliable predictions than a stand-alone model (Fig. 4). In general, the best cut-off seems to be around the middle; i.e., a simple majority vote (here ≥ 6/11) should suffice. To show the combined effect of all positive results, a model trained with maximum set size was compared to an ensemble of models trained with maximum set size, doubled in size by domain-specific data augmentation and incorporating patient-specific windowing (Fig. 5a/b).

**Table 1:** Model performance/reliability measured by SDC/HD with/without curation, per cost function and for set window versus patient-specific window

		Set sizes (SMG/PG)			SDC		HD	
		Train	Validation	Test	SMG	PG	SMG	PG
Data quality								
*Without augmentation*								
Train data	Test data							
Clinical	Clinical	18/18	2/2	4/4	.68 ± .06	.68 ± .05	17.6 ± 1.5	24.7 ± 5.1
Curated	Curated	18/18	2/2	4/4	.66 ± .07	.68 ± .04	23.4 ± 1.3	28.1 ± 4.3
*With augmentation*								
Train data	Test data							
Clinical	Clinical	90/90	2/2	4/4	.67 ± .06	.69 ± .06	13.6 ± 1.3	24.8 ± 5.3
Curated	Curated	90/90	2/2	4/4	.67 ± .07	.69 ± .04	12.0 ± 1.5	21.8 ± 4.6
Cost functions								
SDC		90/90	10/10	20/20	.71 ± .06	.71 ± .06	6.9 ± 1.5	17.3 ± 6.4
SDC(0.5)		90/90	10/10	20/20	.71 ± .06	.71 ± .06	9.0 ± 3.0	17.4 ± 6.8
SDC(0.05)		90/90	10/10	20/20	.70 ± .06	.71 ± .06	6.6 ± 1.6	16.6 ± 7.0
SDC + HD		90/90	10/10	20/20	.70 ± .05		7.6 ± 2.4	
Patient-specific windowing								
Set window		940/1024	94/114	188/227	.86 ± .07	.85 ± .05	4.5 ± 1.9	8.1 ± 3.8
Patient-specific window		940/1024	94/114	188/227	.87 ± .05	.87 ± .04	4.1 ± 1.6	7.7 ± 3.8

## Discussion

**Fig. 6:**
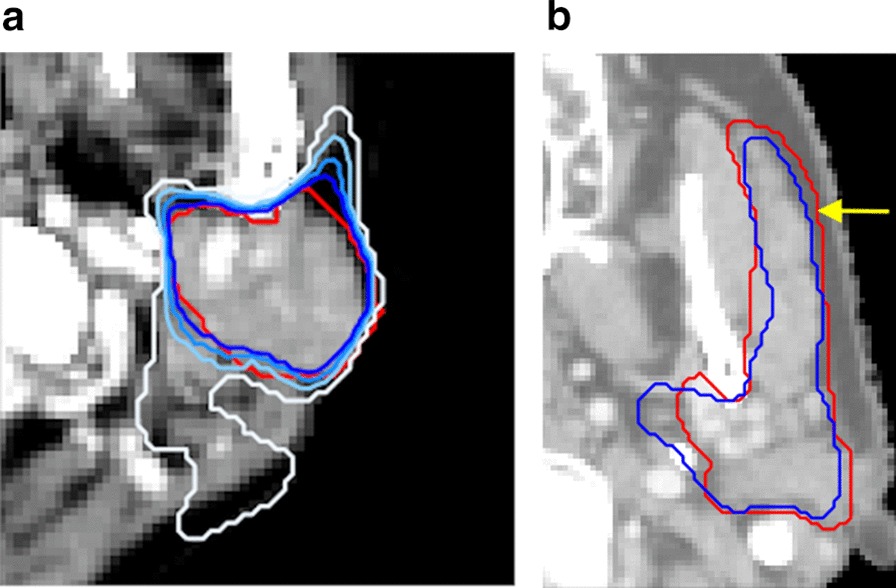
**a** Illustrative example of the effect of using an ensemble for an oddly shaped PG; different cut-off levels depicted by shades of blue (low = light, high = dark), clinical delineation is in red. **b** An inaccuracy (indicated by the yellow arrow) in the clinical contour (red) causing the SDC to be lower than when the DL contour (blue) would have been compared to the actual ground-truth

Several strategies designed to increase model performance and reliability for DL-based salivary gland delineation were explored to facilitate its adoption in clinical practice. Consistent with the work of others, we observed a positive effect from increasing the amount of training data [16]. Data augmentation, patient-specific HU windowing and an ensemble of models also delivered improvements, although the effect of data augmentation was not completely stable. One possible explanation could be the random nature of the augmentation methods we used. Part of the effect of data augmentation might simply be due to an increase in the amount of data, so that more training cases are processed before the early stop is triggered. This may have caused some models that would have otherwise stopped training prematurely to be able to move past flat optimization landscapes. Nevertheless, early stopping was needed to limit total training time and avoid overfitting. While various traditional methods of data augmentation exist [12], domain-specific augmentation is usually achieved with the use of generative adversarial networks [17, 18]. The downside of such networks is that they pose a separate DL problem on their own, whereas the method used here does not. The results of the patient-specific HU windowing may also be important for other imaging applications where the specific task may benefit from having more optimal contrast. Ensembles have been found to work better than stand-alone models before [19], but often these approaches change substantial parts of the model (e.g., architecture or hyperparameters) to obtain different models, while the simple approach used here only needs multiple runs/parameter initializations. Figure 6a shows an illustrative example of how ensemble methods can help make the overall DL framework more reliable. A single model (shown in white) is thrown off by the oddly shaped PG, but when a majority vote over multiple models is held, the DL contour improves significantly.

We did not observe any effect from training on curated data, nor from using different cost functions. The lack of effect from curation was unexpected [20], and might be due to the small set size used in this preliminary analysis. In addition, it is possible that training was harder with the curated data, since, in general, it consisted of more complex contours than the clinical data (Fig. 2e/f). We can quantify this difference in complexity by the mean of the contour circumference divided by its area for all slices (https://matplotlib.org), resulting in values of 0.35/0.55 for clinical/curated, with lower being smoother and higher more complex. Complex features are harder to learn and so learning curves may be different for curated and clinical data. It is also possible that a larger model architecture is necessary for a curated-only dataset, to adequately capture more (and more complex) features. Further research is needed to test these hypotheses. Although other work [21] has found a positive effect of curation the clinical and curated datasets comprised different patients, decreasing the validity of the results. None of the alternative cost functions provided better results than the standard SDC. Apparently, training is not hindered by the small influence that surface errors have on the cost when using the standard SDC. Whenever alternative cost functions are explored, it is usually to deal with highly unbalanced data [e.g., 22], which we don’t have with our single-class approach.

The effects of all strategies seemed to diminish when the model performance was already quite good. For example, when the entire dataset was used, applying an ensemble of PG models only increased average performance by + 0.01 compared to + 0.04 for a set size of 120 cases. This could help to explain why, to the best of the authors’ knowledge, there is no literature reporting SDCs above 0.9 for SMG/PG segmentation. Inter-/intra-observer variability and the inaccurate data associated with it, may put an upper limit on the achievable SDC. For instance, if part of the mandible is included in some PG contours in the train set whilst not in others, the model learns to average over it. If both types of patients are then present in the test set as well, the model’s prediction can never accurately match the clinical contour in all cases making it very unlikely that an average SDC of 1 will ever be possible. Nonetheless, model reliability still seemed to increase, even if base performance was already quite good. This is important for clinical implementation, because clinicians will more often be able to use the contours without having to edit them. We expect that these considerations about the need for high-quality data apply to all applications of deep learning for segmentation, both within the biomedical domain and outside of it. However, we want to be careful extrapolating our other findings to various forms of DL-based radiotherapy segmentation, because effects may well differ between structures due to variation in size, density and contrast with surrounding tissues. For instance, the result of using the strategies applied in this study may be different for much larger and better visible structures like the lungs or much smaller structures like the optic chiasm.

The SDC is the most prevalent outcome metric in biomedical segmentation. However, it can be influenced by multiple factors. DL models can be influenced by randomly splitting the dataset into training and test sets, random weight initialization, random dropout and randomness of the training sequence. Between studies, differences in SDCs can be influenced by the quality of the contours (see above and Fig. 6b), patient characteristics (larger organs give higher SDCs because of the larger proportion of true positives) and imaging characteristics (smaller voxel spacing gives larger organs in voxel space). For all these reasons, inter-study SDC comparisons can be problematic, unless for example, a well curated, open-access dataset is used and intra-study variations are accounted for by cross-validation and multiple runs per fold to account for random weight initialization. Even so, effects of randomness are diminished, but never eliminated. These considerations, except for voxel spacing and organ size (not every metric is influenced by structure size), hold for any metric used to evaluate DL models as we have implemented them. Furthermore, SDC alone may not be sufficient to adequately represent model performance, since, for example, the HD is not always low when SDC is high. Therefore, it may be relevant to report HD and SDC.

The main limitation of this work is considered to be the possible influence of random effects on model performance. Even with cross-validation and, where possible, multiple initializations of the same fold, there is no guarantee that results are not influenced by completely random effects. Other limitations include not accounting for an interaction effect between number of epochs (which may occasionally have been limited too much by early stopping) and the independent variable in an experiment. Lastly, patient-specific HU windowing was based on the SDC. However, the SDC is not available in situations of de-novo contouring, and so if it is to be used in practice, further research is necessary to map CT-scan characteristics to the ‘best’ HU window for segmentation in that scan.

## Conclusions

In summary, several techniques to increase DL model performance and reliability for salivary gland segmentation were investigated. A positive effect was observed from increasing set size, data augmentation, patient-specific HU windowing and a model ensemble. Generally, effects appeared to become smaller when base-model performance was already quite high. This suggests there may be an upper limit for performance governed by data ‘quality’. Nevertheless, model reliability appears to increase regardless of base performance, implying less post-segmentation editing when these techniques are clinically implemented and facilitating the goal of using DLD for autonomous automated segmentation.

## Data Availability

The imaging data analyzed in this work is not publicly available due to the General Data Protection Regulation in European Union law.

## Abbreviations

OAR: Organ-at-risk
DL: Deep learning
DLD: Deep learning-based delineation
HNC: Head and neck cancer
PG: Parotid gland
SMG: Submandibular gland
SDC: Sørensen–Dice coefficient
ROI: Region of interest
HU: Hounsfield unit
HD: Hausdorff distance
